# Cognitive functioning and nausea as stage-specific drivers of quality of life in breast cancer: a longitudinal network analysis

**DOI:** 10.1186/s12955-026-02525-9

**Published:** 2026-05-07

**Authors:** Furong Chen, Jinxian Feng, Min Xu, Shaoxue Li, Yuen-Shan Ho, Naomi Takemura, Janelle Yorke, Jiaying Li, Zengjie Ye

**Affiliations:** 1 School of Nursing, Guangzhou Medical University, Guangzhou, Guangdong Province China; 2 Affiliated Cancer Hospital and Institute of Guangzhou Medical University, Guangzhou, China; 3 School of Nursing, Faculty of Health and Social Sciences, The Hong Kong Polytechnic University, Hong Kong SAR, People’s Republic of China; 4 The Nethersole School of Nursing, Faculty of Medicine, The Chinese University of Hong Kong, Hong Kong, China

**Keywords:** Breast cancer, Symptom management, Quality of life, Network analysis, Precision nursing

## Abstract

**Objective:**

To map the dynamic trajectory of quality of life (QoL) in newly diagnosed breast cancer patients and identify the central symptom drivers that precipitate global health decline during the peri-treatment period.

**Methods:**

In this longitudinal study, patients newly diagnosed with breast cancer (*n* = 337) were recruited at a comprehensive cancer center in Guangzhou, China. The EORTC QLQ-C30 was administered at diagnosis (T1), discharge (T2), and one-month post-discharge (T3). We utilized Cross-Lagged Panel Network (CLPN) analysis to determine temporal relationships and identify influential “bridge” nodes between time points.

**Results:**

Symptom burden shifted significantly over time; insomnia was dominant at diagnosis, whereas fatigue peaked post-discharge. Network analysis revealed two distinct driver mechanisms. During the transition from diagnosis to treatment (T1–T2), Cognitive Functioning emerged as the strongest predictor of subsequent symptom deterioration (Out-Expected Influence [EI] = 0.366). Conversely, in the acute recovery phase (T2–T3), Nausea/Vomiting replaced cognitive issues as the central driver (Out-EI = 0.517), acting as the primary gateway to global health decline.

**Conclusion:**

The drivers of QoL deterioration evolve rapidly during the peri-treatment phase. Cognitive dysfunction at diagnosis serves as an early temporal predictor for somatic symptoms, suggesting that clinical management must shift from reactive symptom control to proactive cognitive prehabilitation at the point of diagnosis.

**Supplementary information:**

The online version contains supplementary material available at 10.1186/s12955-026-02525-9.

## Introduction

With five-year survival rates for breast cancer now exceeding 80% due to multimodal advancements [1, 2], the clinical imperative has shifted from survival extension to the preservation of Quality of Life (QoL) [3]. However, this success comes at a cost. The peri-treatment period, spanning diagnosis, surgery, and adjuvant therapy, remains a critical window of vulnerability [4, 5]. Unlike long-term survivors with established coping mechanisms, newly diagnosed patients face an immediate convergence of physiological trauma and existential distress [6, 7]. Empirical evidence suggests that the symptom burden is highest during this acute phase [8], often establishing a persistent “symptom load” that degrades long-term survivorship [9].

Current management strategies, however, remain reactive and fragmented [10, 11]. The standard of care often treats symptoms such as insomnia or nausea in isolation, neglecting the well-established concept of symptom clusters, concurrent, synergistic conditions that share underlying biological or behavioral mechanisms [12–15]. Although this concept is widely recognized, most existing research employs cross-sectional designs that only capture symptom correlations at a single time point. These static assessments fail to reveal the directionality or the temporal evolution of symptoms [13]. Clinicians are left asking: Does sleep disturbance trigger fatigue, or are both downstream effects of a central cognitive driver? [16].

Understanding these temporal mechanisms is crucial for developing precision nursing interventions [16, 17]. If clinicians can identify the central driver nodes that propel quality-of-life deterioration over time, interventions can be strategically targeted to interrupt these critical pathways [18, 19]. Network analysis, particularly cross-lagged panel network (CLPN) models, offers a distinct advantage by mapping these complex dynamics [16, 20]. Unlike traditional regression models, CLPN quantifies the predictive influence of one symptom on another across time points [21], transforming static data into core targets for intervention [22, 23].

Therefore, this study utilizes longitudinal network analysis to: (1) map the peri-treatment symptom trajectory; (2) isolate the bridge nodes that predict stage-specific deterioration; and (3) provide evidence for targeted nursing interventions that disrupt the symptom cascade at its source.

## Materials and methods

### Study design, participants, and sample size calculation

A prospective longitudinal observational study was conducted using convenience sampling to recruit participants from the “Be Resilient to Breast Cancer (BRBC)” program. Data collection spanned from March to November 2025. Participants were recruited using consecutive sampling to minimize selection bias. The inclusion criteria were strictly defined to ensure a homogeneous sample of newly diagnosed patients: (1) primary diagnosis of breast cancer confirmed by pathology; (2) hospitalization for initial surgical or adjuvant treatment; (3) age ≥ 18 years; and (4) full consciousness with the ability to describe symptoms and complete questionnaires. Exclusion criteria included participation in concurrent drug trials, an expected survival of ≤6 months, or severe non-oncological comorbidities (e.g., cognitive impairment, acute psychiatric disorders) that could confound symptom assessment.

Treatment exposure data were systematically collected from electronic medical records. All 337 participants underwent surgical intervention as the primary inpatient procedure (T1–T2). Of these, 52.8% (*n* = 178) also received the first cycle of adjuvant chemotherapy prior to T2 discharge (primarily Adriamycin and Cyclophosphamide-based regimens [64.1%] or taxane-based protocols [15.4%]), while 47.2% (*n* = 159) were discharged post-surgery with adjuvant chemotherapy scheduled as outpatient treatment. By the T3 assessment (one-month post-discharge), 78.9% (*n* = 266) had initiated or completed at least one cycle of outpatient adjuvant chemotherapy.

The target sample size was determined with the goal of achieving stable network edge estimation and reliable centrality metric rankings. Network stability was empirically evaluated via non-parametric bootstrapping (1,000 iterations) and correlation stability (CS) coefficients [24], as detailed in Sect. "Statistical analysis". The final sample of 337 participants provided adequate stability for centrality estimates (CS coefficients > 0.50 for all key metrics), exceeding the recommended threshold of 0.25 [25].

### Data collection

Data collection was stratified to capture dynamic shifts in symptom burden across the peri-treatment trajectory. The baseline assessment (T1) occurred at admission and the second assessment (T2) at discharge, both conducted via face-to-face paper-based questionnaires by trained research assistants. The final assessment (T3) was conducted one-month post-discharge (±1 week) via WeChat-based questionnaire platform (Questionnaire Star), representing the early recovery phase.

These time points were strategically selected based on the clinical trajectory of breast cancer treatment and previous longitudinal studies [26]. The pre-treatment baseline (T1) captures the patient’s initial symptom burden before therapeutic intervention. The discharge timepoint (T2) represents the acute post-treatment phase when treatment-related side effects typically peak [27]. The one-month post-discharge assessment (T3) corresponds to the early recovery period when acute symptoms begin to stabilize and patients transition to outpatient care, allowing for assessment of persistent versus transient symptom patterns [28].

### Measures

#### Demographic and clinical characteristics

Comprehensive data regarding age, education level, marital status, household income, tumor stage, and relevant comorbidities [29, 30] were collected from electronic medical records and verified via patient self-reports.

#### Quality of life assessment (network nodes)

The Chinese version of the European Organization for Research and Treatment of Cancer Quality of Life Questionnaire Core 30 (EORTC QLQ-C30) was employed as the primary outcome measure [31]. This validated instrument comprises 30 items structured into 5 functional scales, 3 symptom scales, 6 single-item symptoms, and a global health status scale. For network analysis, these 15 subscales were treated as individual nodes. Raw scores were linearly transformed to a 0–100 scale following standard scoring algorithms. To ensure interpretability within the network model, scores were standardized where necessary. For functional scales, higher scores indicate better functioning, whereas for symptom scales, higher scores indicate greater symptom severity.

### Statistical analysis

The statistical analysis comprised three main components: (1) descriptive and repeated measures analysis using IBM SPSS 28.0, (2) Cross-Lagged Panel Network (CLPN) modeling using R software (version 4.4.2) with *glmnet* and *qgraph* packages [23, 32], and (3) network centrality analysis and stability assessment. Statistical significance was set at *α* = 0.05 for all analyses.

Descriptive statistics were calculated for all variables, with continuous variables presented as mean ± standard deviation and categorical variables as frequencies and percentages. Repeated Measures Analysis of Variance (ANOVA) were performed to compare quality of life scores across the three time points (T1, T2, T3), with Bonferroni correction applied for post-hoc pairwise comparisons.

The CLPN was estimated following a node-wise regression approach: for each of the 15 QLQ-C30 subscale scores at time *t* + 1, a separate LASSO-penalized linear regression was estimated using all 15 subscale scores at time t as simultaneous predictors, yielding a 15 × 15 cross-lagged coefficient matrix per transition (T1→T2 and T2→T3). In our CLPN models, we retained the original QLQ-C30 scoring conventions without reverse-coding. All subscale scores were first transformed to a 0–100 scale per EORTC scoring conventions, then z-standardized within each time point to ensure uniform LASSO penalization. The regularization parameter λ was selected via a two-stage procedure: 10-fold cross-validation generated candidate λ values, and the Extended Bayesian Information Criterion (EBIC, *γ* = 0.5) applied to the full dataset determining the final *λ* for each node regression. Autoregressive paths (nodes predicting themselves across time points) were excluded from centrality calculations to isolate cross-node temporal influence, consistent with methodological guidance for driver/bridge node identification in CLPN analyses [25].

Network centrality metrics were calculated, with Out-Expected Influence (Out-EI) as the primary metric quantifying each node’s cumulative predictive effect on subsequent time points, and Bridge Expected Influence measuring connectivity between functional domains. Network stability was assessed via non-parametric bootstrapping (1,000 iterations), with Correlation Stability coefficient > 0.25 considered acceptable [33]. Edge weight accuracy was evaluated through bootstrapped 95% confidence intervals. Sensitivity analyses were performed by retaining autoregressive paths and excluding Stage IV patients to test the robustness of the primary findings.

## Results

### Demographic characteristics

Of 400 questionnaires distributed, 337 participants completed all three waves of data collection, resulting in a high retention rate of 84.3%. The final cohort was predominantly middle-aged (45–60 years: 43.3%) and married (89.6%). Clinically, the sample reflected a curative intent population, with the majority classified as Stage II (40.7%) or Stage III (38.6%). Full demographic details are presented in Table 1.

**Table 1:** General information of the survey subjects (*n* = 337)

Item	Category	n	Percentage (%)
Age (years)	18 ~ <45	120	35.61
	45 ~ <60	146	43.32
	≥60	71	21.07
Education Level	Primary school or below	57	16.91
	Junior high school	84	24.93
	Senior high school/Vocational	120	35.61
	College/Undergraduate	62	18.40
	Master’s degree or above	14	4.15
Marital Status	Married	302	89.61
	Unmarried/Divorced/Widowed	35	10.39
Number of Children	None	26	7.72
	Only child	220	65.28
	Two or more children	91	27.00
Employment Status	Employed	197	58.46
	Retired	73	21.66
	Unemployed	67	19.88
Residence	Urban	183	54.30
	Rural	154	45.70
Monthly Income (CNY)	<3,000	65	19.29
	3,000 - 4999	162	48.07
	5,000 - 10,000	82	24.33
	>10,000	28	8.31
Comorbidities	Yes	53	15.73
	No	284	84.27
Pathological Type	Invasive ductal carcinoma	127	37.68
	Invasive lobular carcinoma	133	39.47
	DCIS/with microinvasion	52	15.43
	Others	25	7.42
Tumor Stage	I	32	9.50
	II	137	40.65
	III	130	38.58
	IV	38	11.27
Tumor Differentiation	Well differentiated	146	43.32
	Moderately differentiated	140	41.54
	Poorly differentiated	51	15.14

### Longitudinal trajectories of quality of life

Repeated measures ANOVA revealed distinct temporal phenotypes across the peri-treatment period (Table 2). Insomnia scores were highest at T1 (mean ± SD: 68.30 ± 23.28) and decreased significantly at T2 and T3 (*p* < 0.001). Conversely, Fatigue followed an inverse trajectory, escalating from admission (T1: 44.09 ± 24.44) to peak severity during the recovery phase at T3 (74.13 ± 19.95; *p* < 0.001). Functional domains showed a robust recovery pattern. Cognitive and Emotional Functioning were lowest at diagnosis (T1) but showed significant linear improvement over time (*p* < 0.001 for both). Similarly, Physical Functioning significantly improved post-discharge, reflecting the restoration of somatic capacity (*p* < 0.001).

**Table 2:** Quality of life of newly diagnosed breast cancer patients at different time points (*n* = 337)

Scale/Item	T1	T2	T3	F	Pairwise Comparison
Physical functioning	72.32 ± 21.90^a^	74.76 ± 15.44^a^	86.17 ± 10.69^b^	88.71^***^	T1≈T2 < T3
Role functioning	65.92 ± 19.38^a^	68.20 ± 14.44^a^	81.21 ± 13.95^b^	100.34^***^	T1≈T2 < T3
Emotional functioning	60.05 ± 16.80^a^	73.27 ± 11.09^b^	78.49 ± 10.08^c^	194.46^***^	T1 < T2 < T3
Cognitive functioning	51.93 ± 17.35^a^	73.39 ± 14.86^b^	82.10 ± 14.75^c^	361.54^***^	T1 < T2 < T3
Social functioning	66.31 ± 17.44^a^	76.81 ± 17.59^b^	85.21 ± 15.10^c^	128.98^***^	T1 < T2 < T3
Fatigue	44.09 ± 24.44^a^	55.37 ± 21.46^b^	74.13 ± 19.95^c^	233.65^***^	T1 < T2 < T3
Nausea and vomiting	27.07 ± 19.93^a^	27.00 ± 13.29^a^	20.21 ± 10.88^b^	27.87^***^	T1≈T2 > T3
Pain	14.29 ± 16.09^a^	20.08 ± 17.22^b^	12.02 ± 14.25^a^	26.04^***^	T1 < T2 > T3
Dyspnea	36.10 ± 18.91^a^	27.25 ± 16.52^b^	15.88 ± 13.95^c^	169.74^***^	T1 > T2 > T3
Insomnia	68.30 ± 23.28^a^	22.55 ± 18.70^b^	12.56 ± 17.17^b^	723.65^***^	T1 > T2 > T3
Appetite loss	27.70 ± 21.77^a^	26.90 ± 19.11^a^	21.07 ± 16.50^b^	14.15^***^	T1≈T2 > T3
Constipation	16.02 ± 21.37^a^	19.88 ± 20.50^a^	9.00 ± 15.69^b^	31.19^***^	T1≈T2 > T3
Diarrhea	5.74 ± 13.85^a^	10.88 ± 17.26^b^	5.44 ± 12.86^a^	15.59^***^	T1 < T2 > T3
Financial difficulties	12.27 ± 20.44^a^	14.94 ± 17.76^a^	8.80 ± 14.94^b^	11.41^***^	T1≈T2 > T3
Global health status	29.08 ± 23.11^a^	33.83 ± 24.19^b^	39.66 ± 29.98^c^	21.04^***^	T1 < T2 < T3

**Note:** T1: At admission; T2: At discharge; T3: One month after discharge. *F*: Test statistics for repeated measures analysis of variance; ^***^p < 0.001. Different superscript letters (a, b, c) indicate statistically significant differences between time points (*p* < 0.05, Bonferroni corrected), while the same superscript indicates no significant difference. For example, in physical functioning, “a” at T1 and T2 indicates no significant difference between these time points, while “b” at T3 indicates a significant difference compared to T1 and T2

### Dynamic cross-lagged panel network models

**Fig. 1:**
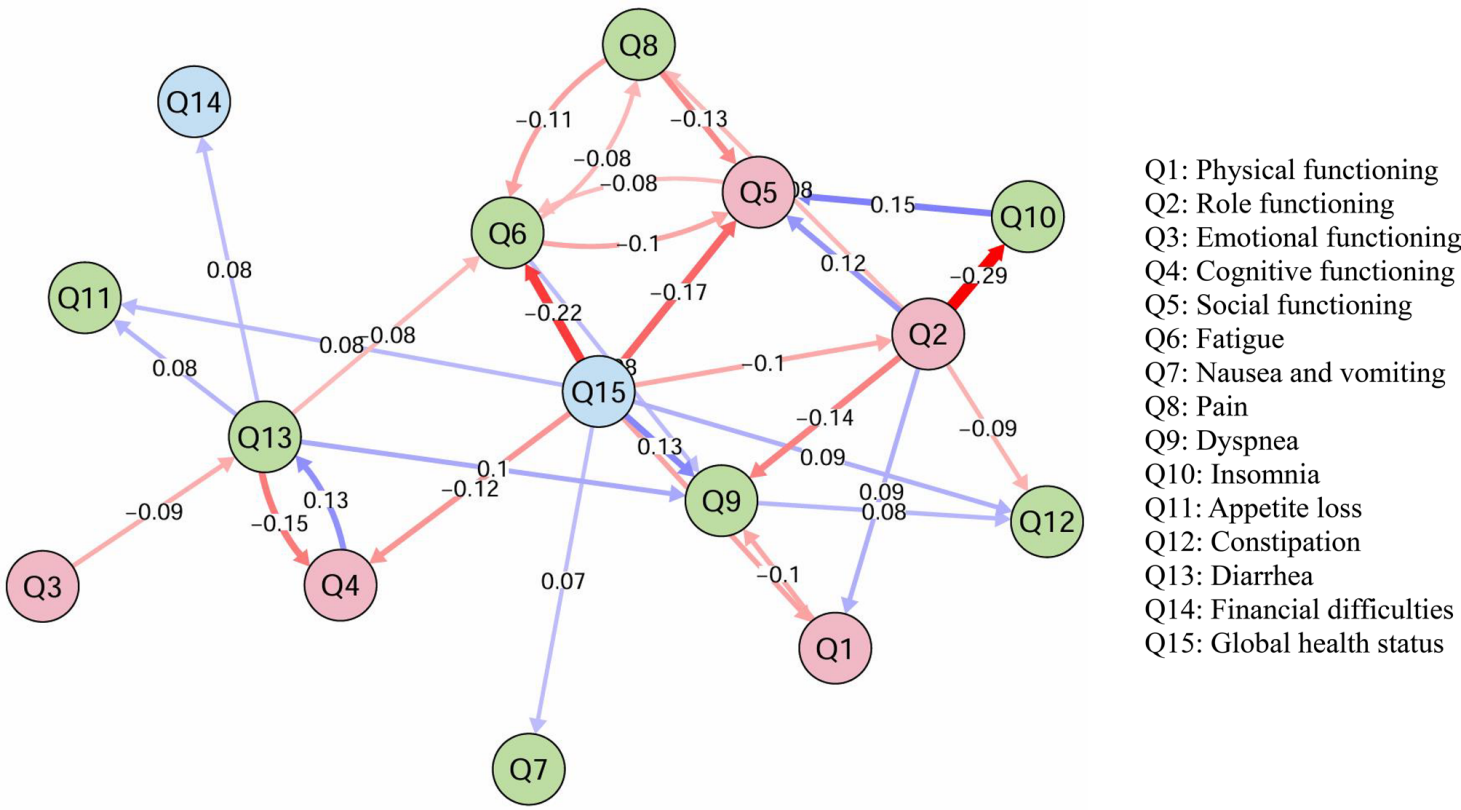
Results of the temporal dynamic network analysis of quality of life from T1 to T2 in patients with newly diagnosed breast cancer. *Note*: Q1: physical functioning; Q2: role functioning; Q3: emotional functioning; Q4: cognitive functioning; Q5: social functioning; Q6: Fatigue; Q7: nausea and vomiting; Q8: Pain; Q9: Dyspnea; Q10: Insomnia; Q11: appetite loss; Q12: Constipation; Q13: Diarrhea; Q14: financial difficulties; Q15: global health status. Edge interpretation: negative edges from functional nodes (Q1–Q5, Q15) to symptom nodes (Q6–Q14) indicate protective temporal relationships; positive edges between symptom nodes indicate propagation relationships. See Sect. "Statistical analysis" for full interpretation framework

**Fig. 2:**
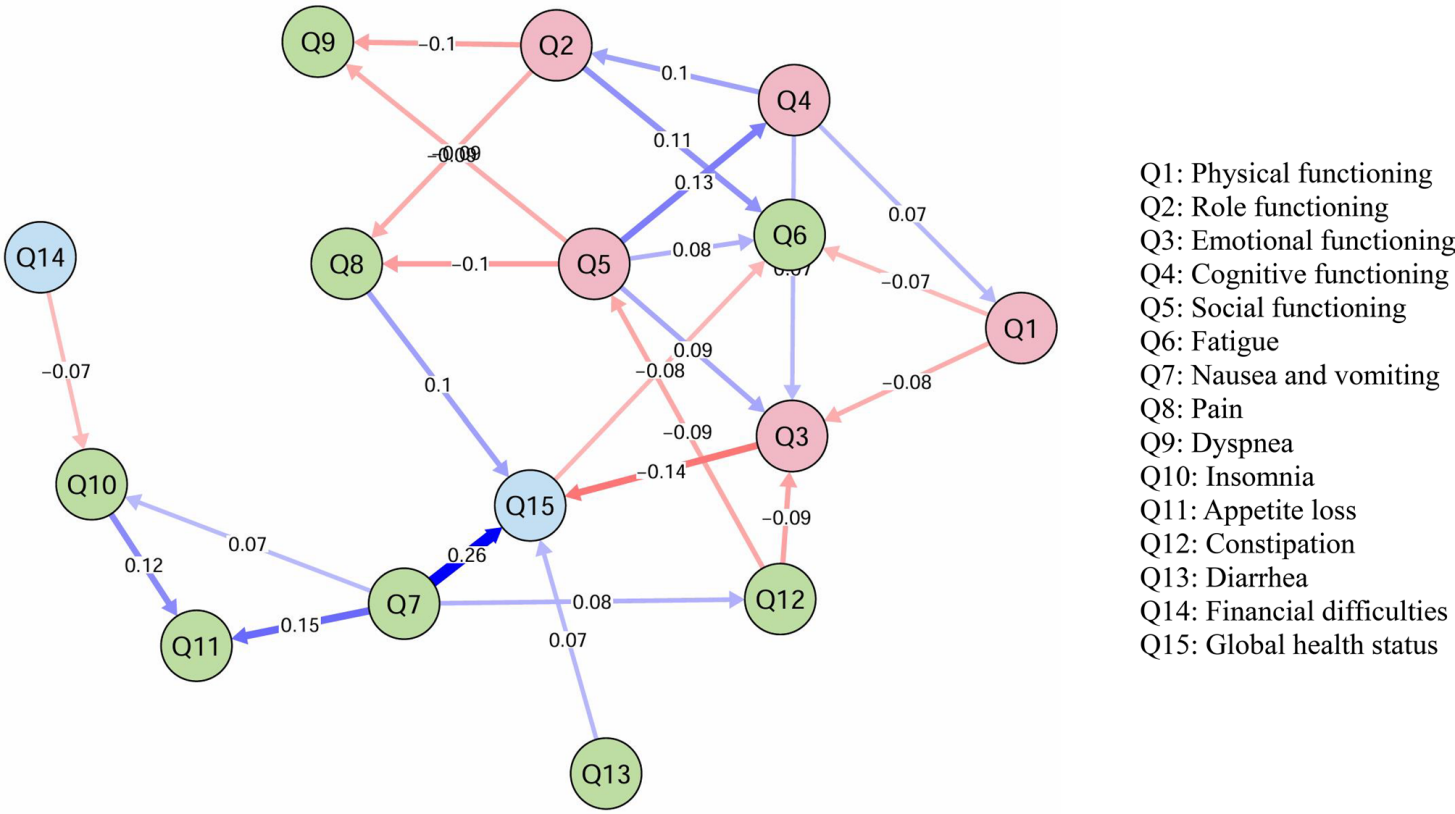
Results of the temporal dynamic network analysis of quality of life from T2 to T3 in patients with newly diagnosed breast cancer. [note same as Fig. 1]

CLPN models, regularized with LASSO, were estimated separately for the T1→T2 and T2→T3 transitions (Figs. 1 and 2). After regularization and removal of autoregressive paths to focus on temporal predictions, the T1→T2 and T2→T3 networks retained 48 and 36 non-zero cross-lagged edges, respectively. Full path coefficient matrices are provided in Supplementary Tables 1 and 2.

In the T1→T2 network, cross-lagged edges were predominantly negative and originated primarily from functional scales and global health status. The strongest edges included role functioning at T1 → insomnia at T2 (*β* = −0.291), global health status at T1 → social functioning at T2 (*β* = −0.221), and global health status at T1 → fatigue at T2 (*β* = −0.220, consistent with prior estimates). Additional prominent negative edges from global health status at T1 included predictions to cognitive functioning (*β* = −0.173) and physical functioning (*β* = −0.120). Other notable edges involved cognitive functioning at T1 → multiple downstream items (*β* range −0.102 to −0.145) and role functioning at T1 → pain-related items (*β* = −0.143).

In the T2→T3 network, nausea/vomiting at T2 emerged as a central driver. It showed strong predictive associations with multiple downstream nodes. Specifically, higher intensity of nausea/vomiting at T2 significantly predicted increased appetite loss (*β* = 0.154) and insomnia (*β* = 0.072) at T3. A strong predictive path was also observed from nausea/vomiting to global health status (*β* = 0.265). Functional scales in this phase showed fewer and weaker connections compared to the T1→T2 transition.

### Centrality and network stability

**Fig. 3:**
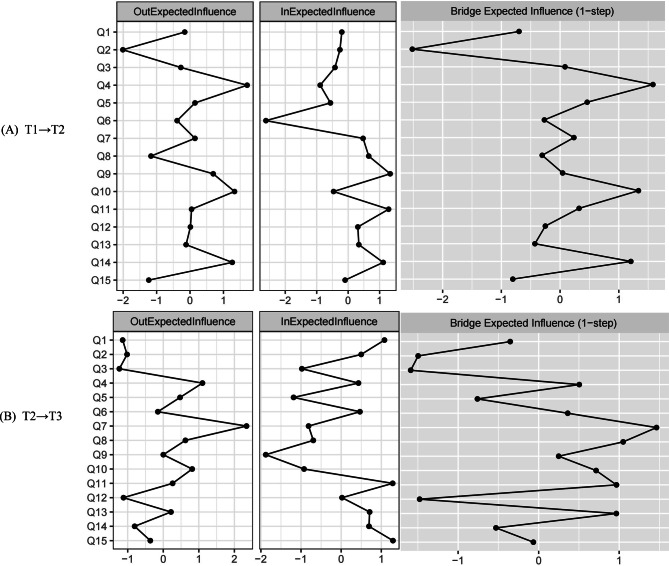
Centrality and bridge centrality estimates of quality-of-life symptoms in newly diagnosed breast cancer patients

Centrality metrics (Out-Expected Influence, Out-EI) quantified the predictive power of specific nodes (Fig. 3). In the T1→T2 network, cognitive functioning exhibited the highest out-expected influence (Out-EI = 0.366), followed by role functioning (Out-EI = 0.28) and global health status (Out-EI = 0.210). For in-expected influence, dyspnea showed the highest value (In-EI = 0.249), followed by insomnia (In-EI = 0.180). In the T2→T3 network, nausea/vomiting had the highest out-expected influence (Out-EI = 0.517), followed by fatigue (Out-EI = 0.320) and appetite loss (Out-EI = 0.250). Global health status exhibited the highest in-expected influence (In-EI = 0.226), followed by fatigue (In-EI = 0.200).

### Network stability

**Fig. 4:**
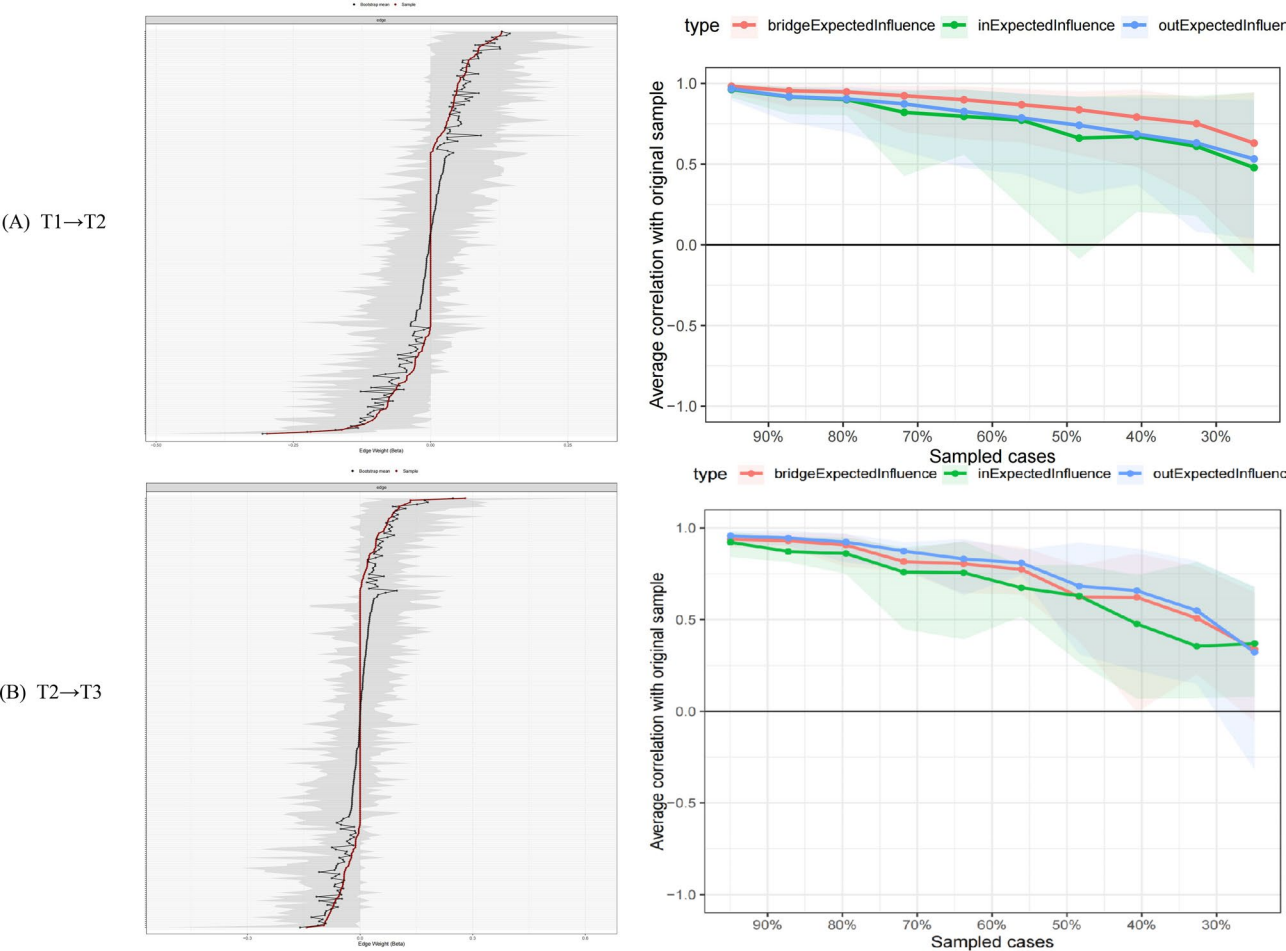
Accuracy and stability analysis of the quality-of-life network in newly diagnosed breast cancer patients

Bootstrap-based stability assessment confirmed the robustness of the network estimates. In the T1→T2 network, CS coefficients were: Out-EI = 0.75, In-EI = 0.67, and Bridge-EI = 0.52, all exceeding the recommended threshold of 0.25. In the T2→T3 network, CS coefficients were: Out-EI = 0.72, In-EI = 0.60, and Bridge-EI = 0.54. These values indicate that the centrality rankings remained stable even after randomly removing up to 70–75% of cases. Regarding edge weight precision, bootstrapped 95% confidence intervals for the top five edges in each network showed minimal overlap, supporting the interpretability of differences in edge strength between the highest-weight paths (Fig. 4).

### Sensitivity analysis

As shown in Supplementary Table 3, the ranking of driver nodes is entirely consistent regardless of whether autoregressive paths are included: Cognitive Functioning remains the top-ranked node in the T1→T2 network, and Nausea and Vomiting remains the top-ranked node in the T2→T3 network. The absolute Out-EI values are slightly attenuated when AR paths are retained, but the ordinal ranking is unchanged. This sensitivity analysis provides additional confidence that our key conclusions are robust to this modeling choice. In addition, we conducted the requested sensitivity analysis excluding Stage IV patients (*n* = 299 remaining). The results were largely consistent with the full-sample analysis. In the T1→T2 network, Cognitive Functioning remained the top-ranked Out-EI node (Out-EI = 0.351). In the T2→T3 network, Nausea/Vomiting remained the primary driver (Out-EI = 0.498).

## Discussion

Cancer patients experience complex symptom interactions that evolve throughout treatment. Using Cross-Lagged Panel Network (CLPN) analysis, we examined how symptoms influence each other over time rather than simply measuring their severity. Our findings reveal that both symptom patterns and their underlying relationships change dramatically across treatment phases. Initially, cognitive functioning serves as a key predictor of physical symptom development during hospitalization. However, following discharge, nausea and vomiting become the primary drivers of symptom clustering during recovery. This temporal shift indicates that effective symptom management requires stage-specific interventions rather than uniform approaches throughout treatment.

A key innovation of this study is the identification of cognitive functioning at T1 as the core driver of the symptom network. Traditionally, cognitive decline in cancer patients is termed “chemobrain” and is viewed primarily as a toxic side effect of chemotherapy [34, 35]. In contrast, our finding that T1 cognitive status, which was assessed prior to or at the very beginning of systemic treatment, strongly predicts T2 somatic symptoms supports the existence of a cognitive-somatic temporally ordered symptom propagation pattern. This phenomenon can be explained through the lens of the Conservation of Resources (COR) theory [36]. The diagnosis shock imposes an immense cognitive load that rapidly depletes the psychological resources of the patient and impairs executive functions such as information processing and emotional regulation [37]. This cognitive depletion acts as an antecedent vulnerability [36]. Patients with lower cognitive resilience at diagnosis may suffer from executive failure, leaving them unable to engage in adaptive health behaviors (e.g., sleep hygiene, stress management) [38]. This failure mechanistically precipitates the collapse in social and physical functioning observed at T2. Crucially, this finding challenges the conventional clinical view that physical symptoms are the primary cause of cognitive decline [39]; instead, our data suggest that early cognitive vulnerability serves as the early temporal predictor for the somatic symptom cluster.

In the transition to the recovery phase (T2–T3), the central regulatory node shifts from the cognitive domain to the somatic domain, with Nausea/Vomiting emerging as the primary driver. While this likely reflects the biological impact of chemotherapy agents stimulating the release of serotonin and inflammatory cytokines [40], our network analysis uniquely characterizes nausea as a node with the highest temporal predictive influence rather than merely an isolated gastric symptom. The network structure reveals a clear bio-behavioral feedback loop wherein nausea directly triggers a nutritional and sleep-related cascade. Specifically, the strong predictive edges from nausea to appetite loss and constipation suggest that gastric distress leads to reduced fluid and fiber intake. This nutritional deficit, in turn, exacerbates insomnia and degrades global health status [41]. This finding provides a novel mechanistic explanation for why fatigue peaks at T3 despite the cessation of acute hospitalization. The severe fatigue observed in the recovery phase is likely the cumulative downstream result of this unresolved gastrointestinal-sleep feedback loop. Consequently, interventions targeting fatigue alone will remain futile unless the central role of nausea in driving downstream symptoms is effectively blocked.

Based on these stage-specific drivers [42], we propose a network-informed precision nursing model that prioritizes interventions based on dynamic influence rather than symptom severity alone. For the diagnostic phase, clinical management must shift focus from reactive symptom control to proactive cognitive prehabilitation. Although current clinical practice often postpones structured rehabilitation until after active treatment [43], our findings underscore the need to initiate Cognitive Prehabilitation immediately at diagnosis to interrupt the T1–T2 deterioration trajectory. Effective strategies should include cognitive resource conservation, such as using simplified visual aids for patient education to reduce cognitive load, and stress inoculation via brief mindfulness-based stress reduction (MBSR) to preserve executive capacity [44]. Furthermore, patients with low baseline cognitive scores should be screened as high-risk candidates for intensive case management to compensate for their reduced adaptive capacity.

As patients transition to the treatment and discharge phase, the priority must shift to targeted interruption of nausea as the central driver of the post-discharge symptom network [40]. Because nausea drives the downstream cluster of insomnia and appetite loss [45], effectively blocking nausea acts as a key node interruption for the entire post-discharge network. Clinical protocols should emphasize the optimized use of prophylactic antiemetics combined with non-pharmacological adjuncts, such as ginger therapy and acupressure [46]. Nurses should recognize that stabilizing gastrointestinal function is paramount, even if fatigue appears more severe in absolute scores. These findings highlight the potential for network-informed, stage-specific interventions to more effectively mitigate the prolonged symptom burden experienced by breast cancer patients during recovery.

Finally, our models revealed a counter-intuitive positive coefficient from nausea/vomiting to global health status in the T2→T3 network. This is inconsistent with the bivariate direction (T2 nausea/T3 global health) and represents a known statistical phenomenon, the ‘suppressor effect’, arising when highly correlated predictors are modeled simultaneously in regularized regression [25, 47]. This is a statistical artifact of the multivariable modeling context and should not be interpreted causally. Critically, our conclusion that Nausea/Vomiting serves as the primary driver in the T2→T3 network is based on its highest Out-Expected Influence centrality and its consistent predictive edges to multiple clinically coherent downstream nodes (appetite loss, insomnia)—not on this single anomalous coefficient. All clinical interpretations in this study strictly prioritize biologically plausible pathways (e.g., the nausea–appetite–sleep axis) and are supported by Out-EI centrality rather than individual edge weights.

### Limitations

Several limitations merit consideration. The study center is a tertiary comprehensive cancer center serving an urban and peri-urban catchment area in southern China; generalizability to rural settings or healthcare systems with different antiemetic or surgical protocols may be limited. Second, while CLPN elucidates temporal precedence, it cannot confirm causality in the absence of experimental manipulation; accordingly, all findings in this study should be interpreted as reflecting temporal predictive influence rather than established causal mechanisms. Future multi-center studies integrating biological markers, such as cortisol or inflammatory cytokines, are needed to validate the physiological mechanisms underlying these symptom networks. Third, reliance on self-report measures introduces the potential for monomethod bias. Future designs would benefit from incorporating objective metrics to calibrate subjective patient experiences.

## Conclusion

This study demonstrates that the drivers of Quality of Life in breast cancer are not static but evolve through distinct, stage-specific mechanisms. We identified a critical trajectory shift: cognitive functioning serves as an early temporal predictor at diagnosis, while nausea/vomiting serves as the gateway to the post-discharge symptom cluster. These findings challenge the traditional “symptom-reactive” model of care. Instead, clinical practice could pivot toward network-informed precision nursing: initiating cognitive prehabilitation immediately upon diagnosis to preserve executive resources, followed by targeted blockage of the nausea node during treatment. By disrupting these specific “driver nodes,” clinicians can attenuate subsequent symptom propagation patterns before they become entrenched, significantly altering the recovery trajectory for newly diagnosed patients.

## Electronic supplementary material

Below is the link to the electronic supplementary material.

Supplementary Material

## Data Availability

The datasets used and/or analyzed during the current study are available from the corresponding author on reasonable request.
