# Supplementary material for: Cognitive functioning and nausea as stage-specific drivers of quality of life in breast cancer: a longitudinal network analysis
Source: Health Qual Life Outcomes. 2026 May 7;24:63. doi: 10.1186/s12955-026-02525-9 (PMC13151383; doi:10.1186/s12955-026-02525-9)
Supplement: Supplementary file 1 — Supplementary Material [file 12955_2026_2525_MOESM1_ESM.docx]

**Supplementary Table 1. Path coefficient matrix of cross-lagged network from T1 to T2 (after removing autoregressive path)**

|  | **Q1** | **Q2** | **Q3** | **Q4** | **Q5** | **Q6** | **Q7** | **Q8** | **Q9** | **Q10** | **Q11** | **Q12** | **Q13** | **Q14** | **Q15** |
| --- | --- | --- | --- | --- | --- | --- | --- | --- | --- | --- | --- | --- | --- | --- | --- |
| **Q1** | 0.066 | 0.060 | 0.046 | 0.042 | -0.047 | -0.069 | -0.001 | -0.100 | 0.000 | 0.000 | -0.053 | 0.000 | 0.000 | 0.000 | 0.066 |
| **Q2** | 0.000 | 0.052 | 0.045 | 0.119 | 0.000 | 0.000 | -0.082 | -0.143 | -0.291 | -0.053 | -0.086 | -0.069 | 0.000 | -0.059 | 0.000 |
| **Q3** | 0.000 | 0.000 | -0.008 | -0.025 | 0.000 | 0.000 | 0.002 | 0.045 | 0.000 | -0.018 | 0.000 | -0.092 | 0.013 | 0.000 | 0.000 |
| **Q4** | 0.000 | -0.005 | 0.000 | 0.000 | 0.057 | 0.036 | 0.051 | 0.040 | 0.000 | 0.036 | 0.000 | 0.127 | 0.024 | 0.000 | 0.000 |
| **Q5** | -0.043 | 0.000 | 0.000 | 0.000 | -0.076 | 0.000 | 0.035 | 0.063 | -0.004 | 0.000 | 0.000 | 0.000 | 0.038 | 0.000 | -0.043 |
| **Q6** | 0.000 | -0.031 | -0.059 | -0.102 | 0.000 | -0.019 | -0.083 | 0.076 | 0.067 | 0.000 | 0.000 | 0.000 | -0.001 | 0.044 | 0.000 |
| **Q7** | 0.000 | 0.000 | -0.014 | -0.048 | -0.005 | 0.000 | 0.005 | 0.000 | 0.008 | 0.012 | 0.000 | 0.000 | 0.054 | 0.000 | 0.000 |
| **Q8** | 0.000 | -0.066 | 0.038 | -0.132 | -0.106 | 0.000 | 0.000 | -0.013 | -0.017 | 0.000 | 0.010 | 0.000 | 0.000 | 0.000 | 0.000 |
| **Q9** | -0.034 | 0.000 | -0.028 | 0.000 | 0.000 | 0.064 | 0.000 | 0.000 | 0.000 | 0.051 | 0.083 | 0.000 | 0.000 | 0.000 | -0.034 |
| **Q10** | 0.065 | 0.054 | 0.063 | 0.145 | 0.000 | -0.012 | -0.001 | 0.000 | 0.000 | -0.006 | -0.002 | 0.000 | 0.000 | -0.025 | 0.065 |
| **Q11** | 0.000 | 0.000 | 0.000 | 0.017 | -0.069 | 0.000 | 0.000 | 0.000 | 0.033 | 0.000 | 0.000 | 0.009 | 0.000 | 0.000 | 0.000 |
| **Q12** | -0.028 | -0.057 | -0.015 | -0.027 | 0.000 | 0.000 | 0.043 | 0.048 | 0.000 | 0.000 | 0.000 | 0.035 | 0.000 | 0.000 | -0.028 |
| **Q13** | 0.000 | -0.069 | -0.149 | -0.022 | -0.081 | 0.000 | 0.046 | 0.098 | 0.000 | 0.084 | 0.000 | 0.000 | 0.075 | 0.000 | 0.000 |
| **Q14** | 0.000 | 0.000 | 0.000 | 0.070 | 0.000 | 0.001 | 0.070 | 0.000 | 0.047 | 0.052 | 0.000 | 0.026 | 0.000 | 0.000 | 0.000 |
| **Q15** | -0.099 | -0.044 | -0.120 | -0.173 | -0.221 | 0.074 | 0.026 | 0.135 | 0.043 | 0.081 | 0.090 | 0.012 | 0.000 | 0.000 | -0.099 |

**Note:** Q1: Physical functioning; Q2: Role functioning; Q3: Emotional functioning; Q4: Cognitive functioning; Q5: Social functioning; Q6: Fatigue; Q7: Nausea and vomiting; Q8: Pain; Q9: Dyspnea; Q10: Insomnia; Q11: Appetite loss; Q12: Constipation; Q13: Diarrhea; Q14: Financial difficulties; Q15: Global health status

·

**Supplementary Table 2. Path coefficient matrix of cross-lagged network from T2 to T3 (after removing autoregressive path)**

|  | **Q1** | **Q2** | **Q3** | **Q4** | **Q5** | **Q6** | **Q7** | **Q8** | **Q9** | **Q10** | **Q11** | **Q12** | **Q13** | **Q14** | **Q15** |
| --- | --- | --- | --- | --- | --- | --- | --- | --- | --- | --- | --- | --- | --- | --- | --- |
| **Q1** | 0.000 | 0.000 | -0.084 | 0.000 | -0.015 | -0.074 | 0.000 | 0.000 | -0.044 | -0.064 | 0.000 | 0.000 | 0.059 | 0.000 | 0.000 |
| **Q2** | 0.037 | 0.000 | 0.000 | 0.003 | 0.064 | 0.113 | -0.043 | -0.087 | -0.097 | -0.042 | -0.018 | -0.057 | -0.051 | 0.000 | -0.019 |
| **Q3** | 0.064 | 0.000 | 0.000 | 0.009 | 0.000 | 0.000 | -0.066 | -0.038 | -0.068 | 0.000 | 0.000 | 0.000 | 0.000 | 0.000 | -0.142 |
| **Q4** | 0.074 | 0.097 | 0.073 | 0.000 | 0.000 | 0.042 | -0.002 | 0.035 | -0.020 | -0.044 | 0.000 | 0.000 | 0.000 | 0.000 | 0.000 |
| **Q5** | 0.038 | 0.044 | 0.091 | 0.134 | 0.000 | 0.082 | -0.039 | -0.102 | -0.085 | -0.011 | 0.000 | 0.000 | 0.000 | 0.000 | -0.030 |
| **Q6** | -0.012 | 0.000 | 0.000 | 0.000 | 0.000 | 0.000 | 0.000 | 0.000 | 0.000 | 0.000 | 0.000 | 0.000 | 0.000 | 0.000 | 0.000 |
| **Q7** | -0.006 | 0.000 | -0.056 | 0.000 | -0.043 | 0.000 | 0.000 | 0.049 | 0.002 | 0.072 | 0.154 | 0.081 | 0.000 | 0.000 | 0.265 |
| **Q8** | 0.000 | 0.000 | -0.002 | 0.000 | 0.000 | 0.000 | 0.014 | 0.000 | 0.000 | 0.000 | 0.000 | 0.000 | 0.043 | 0.000 | 0.098 |
| **Q9** | 0.000 | -0.013 | -0.027 | 0.000 | 0.000 | 0.000 | 0.000 | 0.000 | 0.000 | 0.043 | 0.000 | 0.000 | 0.007 | 0.010 | 0.000 |
| **Q10** | 0.027 | 0.000 | 0.003 | 0.000 | 0.000 | 0.020 | 0.000 | 0.000 | 0.010 | 0.000 | 0.119 | 0.000 | 0.000 | 0.012 | 0.000 |
| **Q11** | 0.050 | 0.000 | -0.005 | 0.000 | 0.000 | 0.000 | 0.000 | 0.001 | -0.007 | 0.000 | 0.000 | 0.000 | 0.000 | 0.056 | -0.020 |
| **Q12** | -0.047 | -0.027 | -0.095 | -0.057 | -0.091 | -0.026 | 0.000 | 0.025 | 0.065 | 0.000 | 0.000 | 0.000 | 0.016 | 0.019 | 0.000 |
| **Q13** | 0.015 | 0.000 | 0.000 | 0.000 | -0.020 | 0.014 | 0.000 | 0.000 | 0.000 | -0.029 | 0.000 | 0.000 | 0.000 | 0.012 | 0.075 |
| **Q14** | -0.046 | 0.000 | -0.011 | 0.000 | 0.000 | 0.000 | 0.027 | 0.028 | -0.048 | -0.070 | -0.031 | 0.000 | 0.000 | 0.000 | 0.000 |
| **Q15** | -0.004 | 0.000 | -0.023 | 0.000 | -0.065 | -0.077 | 0.000 | 0.000 | 0.012 | 0.019 | 0.000 | 0.000 | 0.059 | 0.021 | 0.000 |

**Note:** Q1: Physical functioning; Q2: Role functioning; Q3: Emotional functioning; Q4: Cognitive functioning; Q5: Social functioning; Q6: Fatigue; Q7: Nausea and vomiting; Q8: Pain; Q9: Dyspnea; Q10: Insomnia; Q11: Appetite loss; Q12: Constipation; Q13: Diarrhea; Q14: Financial difficulties; Q15: Global health status

**Supplementary Table 3. Sensitivity analysis of Top 3 Out-Expected Influence nodes with and without autoregressive (AR) paths.”**

| **Network** | **Rank** | **Without AR paths (original)** | **With AR paths (sensitivity)** |
| --- | --- | --- | --- |
| T1→T2 | 1st | Cognitive Functioning (Out-EI = 0.366) | Cognitive Functioning (Out-EI = 0.341) |
| T1→T2 | 2nd | Role Functioning (Out-EI = 0.280) | Role Functioning (Out-EI = 0.253) |
| T1→T2 | 3rd | Global Health Status (Out-EI = 0.210) | Global Health Status (Out-EI = 0.198) |
| T2→T3 | 1st | Nausea and Vomiting (Out-EI = 0.517) | Nausea and Vomiting (Out-EI = 0.489) |
| T2→T3 | 2nd | Fatigue (Out-EI = 0.320) | Fatigue (Out-EI = 0.305) |
| T2→T3 | 3rd | Appetite Loss (Out-EI = 0.250) | Appetite Loss (Out-EI = 0.231) |
